# Alternative Neuroablative Strategies in Facet‐Mediated Pain: Cryoneurolysis Following Incomplete RFA

**DOI:** 10.1155/crra/1257423

**Published:** 2026-11-09

**Authors:** Junman Kim, Brandon E. Hui

**Affiliations:** ^1^ Department of Radiology Emory University School of Medicine Atlanta Georgia USA emory.edu; ^2^ Philadelphia College of Osteopathic Medicine Suwanee Georgia USA pcom.edu

## Abstract

Lumbar facet arthropathy is a common diagnosis that directly correlates with age and is a condition with no formal treatment. A 46‐year‐old female with chronic right lower back and buttock pain has CT scans which revealed right L4–L5 and L5–S1 facet joint arthrosis. After the patient received two radiofrequency ablation procedures, with the latter being less effective than the first, the patient′s pain was still present. Because of persistent pain, cryoneurolysis was proposed as an alternative intervention. The patient experienced a similar degree of pain relief (pain level 8/10–3/10) as well as a more favorable acute postprocedural recovery. This depicts how cryoneurolysis represents a promising alternative neuroablative strategy for patients with lumbar facet arthropathy who have experienced inadequate relief following radiofrequency ablation.

## 1. Background

Lumbar facet arthropathy is caused by aging, wear‐and‐tear over time, previous spinal injuries, poor posture, obesity, or degenerative disc disease. This can lead to chronic low back pain, which is exacerbated on exertion. One of the more severe complications faced, however, is a decreased range of motion, especially when rotating or extending the back. Extending the back compresses the posterior elements and exerts stress on the facet joint, increasing stress and exacerbating pain. With rotation, this creates shearing forces across the facet joint surface, thus promoting cartilage degeneration and pain. The combination of both extension and rotation increases the risk of back pain significantly. Lumbar facet arthrosis has been shown to present in 57% of individuals, starting as early as 20–29 years old, and in 100% of those who are 60 years old and older; among these individuals, the spinal segment most commonly affected is L4–L5 [1]. The prevalence increases exponentially with age, proving age to be a leading risk factor. Furthermore, this is consistent with cartilage degeneration over time within these spinal segments. From a study consisting of 500 patients with chronic, nonspecific spine pain, 31% of those patients had lumbar facet‐mediated pain [2]. This shows how almost one of three individuals with chronic nonspecific spine pain originates in the lumbar facet joints.

The facet joint is a synovial diarthrodial joint that wears out over time with repetitive loading, resulting in joint space narrowing. As cartilage degenerates, subchondral bone remodels, and osteophytes are formed within. This overall helps to stabilize the joint but can be a contributing factor to stiffness and mechanical irritation. Next, synovial inflammation occurs secondary to cartilage breakdown and stress. Inflammatory mediators sensitize nociceptors within the joint capsule and surrounding soft tissue, resulting in back pain. Medial branches of the dorsal rami innervate lumbar facet joints and transmit pain signals to the brain, making this region a primary target for medial branch blocks, radiofrequency ablation, or cryoneurolysis.

Nonsurgical minimally invasive treatment options consist of facet joint steroid injections, medial branch nerve blocks, radiofrequency ablation, and cryoneurolysis. With facet joint steroid injections, patients receive an injection of corticosteroid and local anesthetic into the facet joint, reducing inflammation in the joint capsule and surrounding tissues. Patients report feeling relief for 1–6 weeks on average, with the longest being up to 3 months [3]. The limitation of steroid injections is that relief is temporary, requiring patients to receive repeated injections to feel long‐term relief. With a medial branch nerve block, an anesthetic is injected around the medial branch nerve that innervates the facet joint, interrupting any pain signaling. Patients tend to feel an improvement in pain control within minutes and provide pain relief for up to 1–3 months if combined with a steroid [4]. However, this technique is mainly used to confirm the facet joint as a source of pain and may require multiple blocks to be administered to feel long‐term analgesia. RFA requires utilizing radiofrequency energy to create a thermal lesion on medial branch nerves, disrupting the pain signaling from the facet joint. This method utilizes thermal coagulation to induce protein denaturation and coagulative necrosis of the targeted nerve. This disrupts the collagenous integrity of the perineurium and epineurium [5]. Furthermore, thermal coagulation impairs axonal regeneration, increasing the probability of uncontrolled nerve damage and neuroma formation. This provides patients with longer term relief of up to 10 months on average [6]. A downside of RFA is that pain reduction is delayed, which may take up to 1–2 weeks postprocedure. With cryoneurolysis, this entails freezing the medial branch nerves, causing Wallerian degeneration that temporarily interrupts any pain signals coming from the facet joint. With Wallerian degeneration, this method preserves the perineurium and epineurium (Sunderland Grade II injury) [5]. This allows the connective tissue framework to follow an organized pathway when regenerating, making the induced neuronal injury reversible. On average, patients reported feeling pain relief for up to 7 months and sometimes even longer, depending on how quickly the nerve regenerates [7]. Although the analgesia window is much shorter than RFA, patients feel pain relief immediately postprocedure compared with those who receive RFA.

This report is based on a patient who received radiofrequency ablation twice, with the latter treatment resulting in an incomplete response. Cryoneurolysis was utilized, which provided adequate symptom relief more quickly after procedure and possibly for a longer period of time. Utilizing cryoneurolysis over traditional radiofrequency ablation may serve as a more optimal option. This case report was prepared and written in accordance with the CARE reporting guidelines and a completed CARE checklist is provided as File S1.

## 2. Case Report

A 46‐year‐old female presents with chronic right lower back and buttock pain. She previously had adequate temporary relief from previous facet joint blocks but returned for lumbar medial branch nerve ablation secondary to recurring pain. Initial lumbar MRI demonstrates severe facet degenerative changes at L4–L5 and L5–S1, including joint space narrowing, facet hypertrophy, and subchondral cystic changes without any significant disc or endplate pathology (Figure 1a,b). On July 7, 2023, the patient successfully received her first treatment of right L4–L5 and L5–S1 medial branch nerve ablation with no complications (Table 1). Before the procedure, the patient rated her pain 8/10 and postprocedure rated her pain 0 with ambulation. On November 27, 2024, she returned with the same recurring episodes of pain, which she rated 8/10 (Table 1). After a significantly less effective second lumbar medial L4 and L5 branch nerve ablation compared with the first, she rated her pain 3/10 with ambulation. This shows that the patient had a diminished response with repeat RFA. On physical examination, the patient demonstrated right lumbar paraspinal tenderness to palpation with lumbar extension and rotation; however lower extremity strength and sensation were intact bilaterally without evidence of any focal neurological deficit. The source of the patient′s pain could be because of an altered lesion, scar formation, or worsening facet arthropathy. In addition, fascial and musculoskeletal sources can be considered as possible pain generators. The main fascial layer that surrounds the lumbar region is the thoracolumbar fascia as it is densely innervated with nociceptors. Patients with low back pain show reduced fascial shear‐strain, increased thickness, and decreased sliding mobility; this produces longer lasting and intense pain when stimulating the muscle or subcutaneous tissue within the thoracolumbar fascia, making this a possible pain generator [8]. Furthermore, muscles, fascia, and ligaments can be a cause of low back pain when it comes to muscle guarding, spasm, atrophy, or referred posterior thigh pain [9]. From this, it was decided that the next step in her plan of care would be cryoneurolysis. The patient is a perfect candidate for medial branch ablation, as shown by a previous L‐spine MRI. MRI depicts no significant disc or endplate pathology but shows an isolated degeneration of the facet degenerative changes at L4–L5 and L5–S1. When seeking a longer pain relief option, the patient returned on May 9, 2025, with 8/10 and underwent CT‐guided cryoneurolysis of the right L4 and L5 medial branch nerves (Table 1). After the patient was sterilely prepped, CT fluoroscopic guidance was utilized, and two Boston Scientific IceSphere probes (17‐gauge) were appropriately positioned at the corner of the superior articular process and transverse process junction at left L4 and L5 (Figure 2). The patient was administered Ketolag 40 mg mixed with 15 cc 0.25% bupivacaine delivered to three spots. Each location was cryoablated utilizing two cycles: 8‐minute freeze and 3 minutes of passive thaw and subsequently a 3‐minute freeze and 3‐minute passive thaw. After successful ablation, the probes were removed, and hemostasis was achieved with manual compression. The patient tolerated the procedure well with no complications. She rated her pain 3/10 with ambulation. The patient was not taking medication before or after cryoneurolysis procedure.

**Figure 1:**
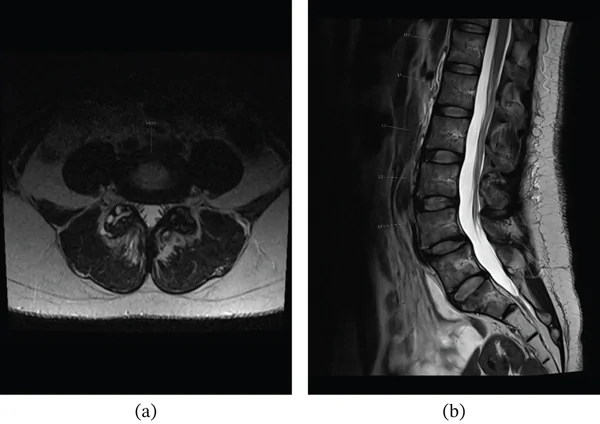
T1WI (a) axial MRI and T2WI (b) sagittal MRI depicting severe facet degenerative changes at L4–L5 and L5–S1. This is demonstrated by joint space narrowing, subchondral cystic changes, and hypertrophy. There is no significant disc/endplate pathology.

**Table 1:** Timeline of diagnostic evaluation, therapeutic interventions, and clinical outcomes has been outlined in 46‐year‐old female with lumbar facet‐mediated lower back pain.

Date	Clinical event/intervention	Clinical findings and outcomes
Prior to July 7, 2023	Diagnostic facet joint block	Temporary pain relief following diagnostic facet joint blocks, supporting facet‐mediated pain as a suspected pain generator.
July 7, 2023	Successful right lumbar medial branch RFA at L4–L5 and L5–S1.	Pain decreased from 8/10 to 0/10 with ambulation immediately following the procedure. No reported complications.
November 27, 2024	Repeat right lumbar medial branch RFA at L4–L5 and L5–S1.	Recurrent right‐sided low back and buttock pain rated 8/10. Following repeat RFA, pain decreased to 3/10 with ambulation with no postprocedural complications.
Prior to May 9, 2025	Clinical reassessment and lumbar MRI evaluation.	MRI demonstrated facet degenerative changes at L4–L5 and L5–S1 without significant disc or endplate pathology.
May 9, 2025	CT‐guided cryoneurolysis of the right L4 and L5 medial branch nerves.	Preprocedural pain was rated 8/10. Postprocedural pain decreased to 3/10 with ambulation and with no immediate complications.
May 2026 (1‐year follow‐up)	Postcryoneurolysis clinical follow‐up.	Patient reports having sustained pain relief and was able to return to the gym without any complications.

**Figure 2:**
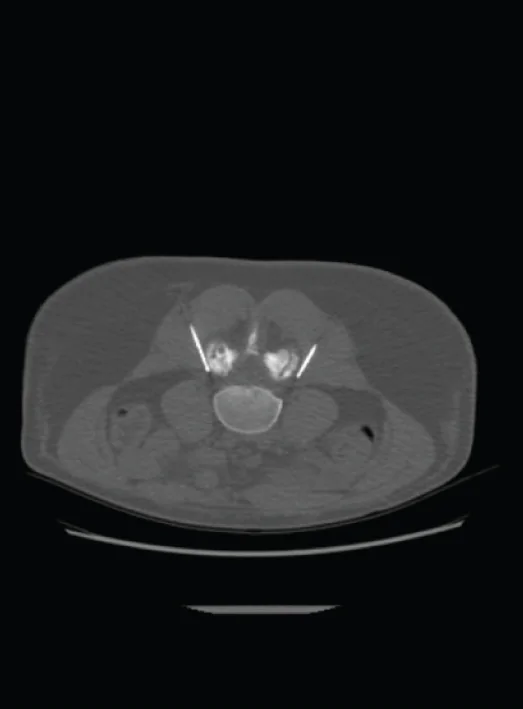
Axial CT‐guided placement of cryoablation probes targeting L4–L5 and L5–S1 medial branch nerves. Probes are positioned posteriorly at the junction of transverse processes and superior articular processes.

It is important to note that the patient had complete relief in 2023, but has had recurring worsening pain postprocedurally, with a consistent 3/10 pain with ambulation (Table 1). After receiving an incomplete RFA, cryoablation was able to achieve a meaningful pain reduction. On follow‐up visit after 1‐year postcryoneurolysis, the patient reports still having good pain relief and is able to go back to the gym without any complications. Furthermore, the procedure was well tolerated and reproducibly effective. This depicts how cryoneurolysis may serve as a viable alternative option for patients who have a history of repetitive RFA treatments.

## 3. Discussion

When utilizing a > 75% pain reduction threshold for at least 6 months or longer, both cooled and traditional RFA were measured to have a ~50% success rate [10]. This shows that approximately one in two patients do not experience substantial relief post‐RFA. Furthermore, this emphasizes how common incomplete RFAs can be. The primary RFA techniques used in medial branch neurotomy consist of conventional and cooled RFA. Conventional RFA utilizes a high‐frequency alternating current which generates heat within the tissue surrounding the electrode tip through ionic friction [11]. This produces a relatively small ellipsoid lesion along the active tip of the cannula, allowing minimal lesion extension beyond the probe tip. Consequently, optimal electrode placement parallel to the nerve is mandatory to provide appropriate pain relief, thus requiring multiple placements of the probe to reliably capture the anatomically variable medial branch [11]. In contrast, cooled RFA utilizes an internal circulation of water through the electrode to dissipate heat. This reduces perielectrode charring and allows energy to propagate farther than the probe tip, creating a larger and more spherical lesion [11]. This causes a significantly larger lesion compared with conventional RFA, providing a more forgiving method when it comes to needle angle and anatomical variability of the medial dorsal nerve [12].

One possible cause of incomplete RFAs could be inadequate lesion size, resulting in the targeted nerve being incompletely ablated. Another cause could be inadequate probe placement. This may result in failing to target the entire nerve, which may cause residual pain in nearby tissues. Furthermore, anatomical variations can serve as a hindrance by making targets seem inaccessible or hard to access. This can complicate the process of creating a lesion.

Despite patients receiving long‐term pain relief in successful RFAs, complications can contribute heavily to inadequate pain relief. Complications can consist of weakness of lumbar paraspinal muscles, dysesthesia, mild neuritis, sensory loss, and new onset of neuropathic pain [13]. More specifically, multifidus denervation is an important consideration following medial branch RFA because the medial branches of the dorsal ramus provide motor innervation to the multifidus muscle. This may result in changes of muscle morphology including atrophy; however, the long‐term clinical significance of this consideration remains uncertain [14]. Compared with cryoablation, this method targets the same nerve and will inevitably cause the same effect. However, because there is preservation of the perineurium and endoneurium, the injury is reversible once the nerve properly heals [15, 16]. A major clinical implication to consider is how this can alter intervertebral joint angles in both flexion and lateral sidebending, promoting tissue stress and disc degeneration [17]. However, studies have not proven whether or not denervation causes permanent destabilizing effect [18]. Furthermore, it has been shown how decreased multifidus activity secondary to post‐RFA has been shown to not necessarily cause any long term functional decline [19]. Because of excessive or inadvertent thermal injury to motor pathways or sensory pathways, this can leave the patient with new symptoms. Additionally, mild neuritis can cause nerve irritation that may be in the form of burning pain. Furthermore, sensory loss or even a new onset of neuropathic pain can further complicate the treatment, requiring additional clinical interventions. This suggests how RFA is not universally effective, failing to meet pain management goals or even worsening patients’ symptoms.

Alternatively, patients who received cryoneurolysis experienced a more favorable safety profile, significant improvements in pain, and functional disability when compared with RFA [20]. Additionally, because cryoneurolysis tends to cause less collateral tissue damage, this can result in fewer reports of postablation neuritis, temporary muscle spasm, and stiffness. However, complications of cryoanalgesia consist of bleeding, bruising, and local skin infections. However, it has been shown that patients who have experienced these complications posttreatment have been able to resolve these symptoms within 1 month [21]. Furthermore, these risks can be eliminated by having the patient regularly follow up with the interventionist while maintaining proper postprocedure care. Permanent nerve damage is rare because cryoneurolysis preserves the nerve′s connective tissue, which allows for regeneration [22]. Although both RFA and cryoneurolysis are reversible, cryoneurolysis preserves the nerve scaffolding, providing a more organized axonal regeneration.

## 4. Conclusion

Patients who have back pain secondary to facet degenerative changes usually start with minimally invasive options. Cryoneurolysis should be the first choice for ablation options after a successful nerve block. Although this is a temporary analgesic solution, there have been minimal postprocedural complications, resulting in significantly better postprocedural relief. Cryoneurolysis is a potentially helpful option that requires more research directly comparing these treatment modalities. This case supports how future practice may shift toward incorporating cryoneurolysis as a main treatment option for facet lumbar pain, especially in patients who may be at a high risk for postablation neuritis.

## Funding

No funding was received for this manuscript.

## Ethics Statement

Institutional Review Board approval was not required for this single‐patient case report in accordance with institutional policy.

## Consent

The patient consented to publication of this report but did not provide a written personal statement.

## Conflicts of Interest

The authors declare no conflicts of interest.

## Supporting information


**Supporting Information** Additional supporting information can be found online in the Supporting Information section. File S1: CARE checklist.

## Data Availability

The data that support the findings of this study are available from the corresponding author upon reasonable request.
