# Supplementary material for: Alternative Neuroablative Strategies in Facet‐Mediated Pain: Cryoneurolysis Following Incomplete RFA
Source: Case Rep Radiol. 2026 Nov 9;2026:1257423. doi: 10.1155/crra/1257423 (PMC13748811; doi:10.1155/crra/1257423)
Supplement: Supplementary file 1 [file CRRA-2026-1257423-s001.docx]

# CARE Checklist — Completed

**Manuscript:** Case Report: Alternative Neuroablative Strategies in Facet-Mediated Pain: Cryoneurolysis Following Incomplete RFA

**Authors:** Hui B, Kim J

**Journal:** Case Reports in Radiology · **Manuscript ID:** 1257423

**Checklist version:** CARE 2013 (Riley DS, Barber MS, Kienle GS, et al. CARE guidelines for case reports: explanation and elaboration document. *J Clin Epidemiol.* 2017;89:218–35)

| # | Topic | Checklist item | Reported | Location in manuscript |
| --- | --- | --- | --- | --- |
| 1 | **Title** | The diagnosis or intervention of primary focus followed by the words “case report” | Yes | Title page. “Case Report: Alternative Neuroablative Strategies in Facet-Mediated Pain: Cryoneurolysis Following Incomplete RFA” |
| 2 | **Key Words** | 2 to 5 key words that identify diagnoses or interventions in this case report, including “case report” | Yes | Following the Abstract. Five keywords: case report; facet joint; radiofrequency ablation; cryoablation; pain relief |
| 3a | **Abstract** | Introduction — what is unique about this case and what it adds to the literature | Yes | Abstract, sentence 6 |
| 3b | **Abstract** | Main symptoms and/or important clinical findings | Yes | Abstract, sentences 2–4 |
| 3c | **Abstract** | Main diagnoses, therapeutic interventions and outcomes | Yes | Abstract, sentences 5–6 |
| 3d | **Abstract** | Conclusion — what are the main “take-away” lessons | Yes | Abstract, sentence 6 |
| 4 | **Introduction** | Brief background summary of this case referencing the relevant medical literature | Yes | Background, full section. Eight references cited |
| 5a | **Patient Information** | De-identified demographic and other patient-specific information | Yes | Case Presentation – Initial Presentation. 46-year-old female; no identifying detail reported; dates reduced to month and year |
| 5b | **Patient Information** | Primary concerns and symptoms of the patient | Yes | Case Presentation – Initial Presentation and Acute Representation; Table 1 |
| 5c | **Patient Information** | Medical, family and psychosocial history including relevant genetic information | Partial* | Case Presentation – Initial Presentation. No relevant family or genetic history was identified. |
| 5d | **Patient Information** | Relevant past interventions and their outcomes | Yes | Case Presentation – Initial Presentation. Facet blocks and both RFAs with pain scores reported; Table 1 |
| 6 | **Clinical Findings** | Describe significant physical examination and important clinical findings | Yes | Case Presentation – Lumbar tenderness, extension/rotation provocation, intact strength and sensation. |
| 7 | **Timeline** | Historical and current information from this episode of care organized as a timeline (figure or table) | Yes | Table 1 |
| 8a | **Diagnostic Assessment** | Diagnostic testing, including physical examination, laboratory testing and imaging | Yes | Case Presentation – MRI and diagnostic blocks reported; quantitative block response and exact timing missing; Figures 1-3. |
| 8b | **Diagnostic Assessment** | Diagnostic challenges — access, financial, cultural | Partial | Case Presentation – Alternative pain generators discussed, but case-specific diagnostic uncertainty/challenges unclear. |
| 8c | **Diagnostic Assessment** | Diagnostic reasoning including other diagnoses considered | Yes | Case Presentation – Facet arthropathy, blocks and exam described; distinguish imaging correlation from diagnostic proof. |
| 8d | **Diagnostic Assessment** | Prognostic characteristics where applicable | Partial | Case Presentation – Recurrent symptoms and prior responses described; formal staging not applicable/identified. |
| 9a | **Therapeutic Intervention** | Types of intervention — pharmacologic, surgical, preventive, self-care | Yes | Case Presentation – Facet blocks, medial branch RFA and cryoneurolysis documented. |
| 9b | **Therapeutic Intervention** | Administration of intervention — dosage, strength, duration | Yes | Case Presentation – CT guidance, probe and cycles detailed; right/left discrepancy and Ketolag 40 mg mixed 15 cc .25% bupivacaine delivered to 3 spots. |
| 9c | **Therapeutic Intervention** | Changes in intervention with rationale | Yes | Case Presentation – Transition after diminished second-RFA response stated; clarify response duration and rationale. |
| 10a | **Follow-up and Outcomes** | Clinician- and patient-assessed outcomes where appropriate | Yes | Case Presentation – Immediate scores and one-year activity/pain relief reported. |
| 10b | **Follow-up and Outcomes** | Important follow-up diagnostic and other test results | Yes | Case Presentation – One-year clinical follow-up reported. |
| 10c | **Follow-up and Outcomes** | Intervention adherence and tolerability, and how this was assessed | Yes | Case Presentation – No medications post cryoneurolysis, good procedural tolerance. |
| 10d | **Follow-up and Outcomes** | Adverse and unanticipated events | Yes | Case Presentation – No immediate complications or reported one-year complications. |
| 11a | **Discussion** | Strengths and limitations in the management of this case | Yes | Discussion – Limitations |
| 11b | **Discussion** | Discussion of the relevant medical literature | Yes | Discussion – Literature on RFA, cryoneurolysis and adverse effects discussed. |
| 11c | **Discussion** | Rationale for conclusions, including assessment of possible causes | Yes | Discussion – Interpretation provided; claims of cryoneurolysis being a superior choice of treatment. |
| 11d | **Discussion** | Primary “take-away” lessons from this case report | Yes | Conclusion |
| 12 | **Patient Perspective** | The patient should share their perspective on the treatment(s) they received | Yes* | Not obtained; stated in the patient perspective section after consent declaration |
| 13 | **Informed Consent** | The patient should give informed consent (provide if requested) | Yes | Ethics Approval and Informed Consent – Institutional Review Board approval was not required for this single-patient case report in accordance with institutional policy |

## Notes

**Item 5c (family history).** Marked partial. Facet-Mediated Pain arises is caused by aging, wear-and-tear over time, previous spinal injuries, poor posture, obesity, or degenerative disc disease. This does not have a recognized heritable basis, so a family history would not be informative.

**Item 12 (patient perspective).** The patient consented to publication of this report but did not provide a written personal statement.

## Declaration

The authors confirm that this case report has been prepared and reported in accordance with the CARE guidelines, and that this checklist accurately reflects the content of the submitted manuscript.

**Corresponding author:** Brandon E. Hui· brandon.e.hui@gmail.com

**Date:** 16 Oct 2026
